# Limitations to Starch Utilization in Barramundi (*Lates calcarifer*) as Revealed by NMR-Based Metabolomics

**DOI:** 10.3389/fphys.2020.00205

**Published:** 2020-03-20

**Authors:** Mariana Palma, Lauren H. Trenkner, João Rito, Ludgero C. Tavares, Emanuel Silva, Brett D. Glencross, John G. Jones, Nicholas M. Wade, Ivan Viegas

**Affiliations:** ^1^Centre for Functional Ecology, Department of Life Sciences, University of Coimbra, Coimbra, Portugal; ^2^CSIRO Agriculture and Food, Queensland Biosciences Precinct, Brisbane, QLD, Australia; ^3^School of Agriculture and Food Science, The University of Queensland, Brisbane, QLD, Australia; ^4^Center for Neuroscience and Cell Biology, University of Coimbra, Coimbra, Portugal

**Keywords:** Asian seabass, ^2^H NMR, metabolomics, aquaculture, hepatic glycogen

## Abstract

Practical diets for commercial barramundi production rarely contain greater than 10% starch, used mainly as a binding agent during extrusion. Alternative ingredients such as digestible starch have shown some capacity to spare dietary protein catabolism to generate glucose. In the present study, a carnivorous fish species, the Asian seabass (*Lates calcarifer*) was subjected to two diets with the same digestible energy: Protein (P) – with high protein content (no digestible starch); and Starch (S) – with high digestible (pregelatinized) starch content. The effects of a high starch content diet on hepatic glycogen synthesis as well as the muscle and liver metabolome were studied using a complementary approach of ^1^H and ^2^H NMR. The hepatosomatic index was lower for fish fed high starch content diet while the concentration of hepatic glycogen was similar between groups. However, increased glycogen synthesis via the direct pathway was observed in the fish fed high starch content diet which is indicative of increased carbohydrate utilization. Multivariate analysis also showed differences between groups in the metabolome of both tissues. Univariate analysis revealed more variations in liver than in muscle of fish fed high starch content diet. Variations in metabolome were generally in agreement with the increase in the glycogen synthesis through direct pathway, however, this metabolic shift seemed to be insufficient to keep the growth rate as ensured by the diet with high protein content. Although liver glycogen does not make up a substantial quantity of total stored dietary energy in carnivorous fish, it is a key regulatory intermediate in dietary energy utilization.

## Introduction

Aquaculture production of carnivorous fish continues to increase worldwide, with barramundi *Lates calcarifer* one such species that is widely cultured in Southeast Asia and Australia (Glencross et al., 2013). Dietary formulations are required to fulfill the species nutritional needs while minimizing dependence on fishmeal protein from wild-sources. To this end, the minimum nutritional requirements of barramundi have been defined (Glencross, 2006; Glencross et al., 2013; Poppi et al., 2017), and a range of terrestrial animal and plant-derived raw ingredients have been evaluated as a partial replacement for fishmeal protein in aquafeeds (Gatlin et al., 2007; Glencross et al., 2012; Glencross B. et al., 2017; Turchini et al., 2019). This has allowed the near complete replacement of both fishmeal and fish oil for this species (Glencross et al., 2016).

Several of the most effective seed sources showed low starch digestibility that was linked with amylopectin content (Glencross et al., 2012), but carbohydrate digestibility was also affected by treatments such as gelatinization or changes during extrusion (Catacutan and Coloso, 1997; McMeniman, 2003). However, the outcomes of partial dietary protein replacement using ingredients that contain highly digestible starch has been disappointing, with increased digestible starch diets causing decreased growth rate (McMeniman, 2003), associated with increased feed conversion ratio (Glencross et al., 2014). Moreover, there were significant effects on whole fish carcass composition, lipid accumulation, and flesh quality (Glencross et al., 2014), as well as decreased protein utilization efficiency as digestible starch increased (Glencross B. D. et al., 2017). These outcomes appear to stem from a poor capacity of this species to incorporate starch carbons into the principal intermediary metabolic pathways of substrate oxidation and biosynthesis. Digestible starch has been demonstrated to be redirected into lipogenesis (Viegas et al., 2019; Williams et al., 2001) however, the precise rate-limiting steps remain uncharacterized. Without this knowledge, the practical replacement of dietary protein will be limited to indigestible starch sources such as barley, wheat, oats, sorghum, and triticale among others (Glencross et al., 2012). Therefore, the purpose of this study was to assess the metabolic limitations of barramundi that underlie poor utilization of highly digestible starch.

**FIGURE 1:**
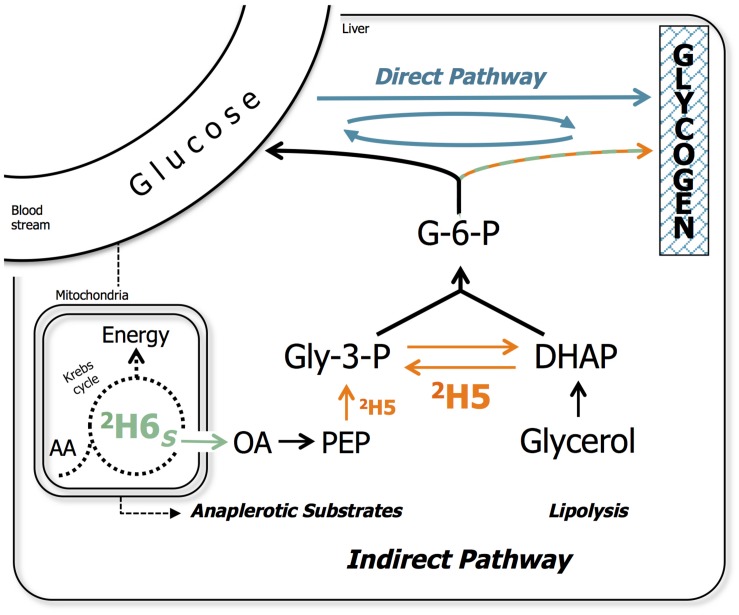
Metabolic model representing the direct and indirect pathways of hepatic glycogen synthesis. Indirect pathway sources include gluconeogenic precursors that are metabolized via the anaplerotic pathways of Krebs cycle (i.e., pyruvate and gluconeogenic amino acids) as well as glycerol from lipolysis. Indirect pathway sources also formally include pyruvate derived from glucose, as indicated by the dashed line. The metabolic pathway sites for ^2^H-enrichment from ^2^H_2_O of glycogen position 5 (^2^H5; in orange), namely enolase and triose phosphate isomerase, and position 6S (^2^H6*_*S*_*; in green), namely fumarase in the Krebs cycle are indicated. The remaining is either preexisting, cycled or from direct pathway (in blue). Several metabolic intermediates were omitted for clarity. G-6-P, glucose-6-phosphate; Gly-3-P, glyceraldehyde-3-phosphate; DHAP, dihydroxyacetone phosphate; OA, oxaloacetate; PEP, phosphoenolpyruvate; AA, amino acids.

Most carnivorous fish species such as European seabass (*Dicentrarchus labrax*) and gilthead seabream (*Sparus aurata*) store excess starch in the form of glycogen (Enes et al., 2011), which is then readily available during fasting (Enes et al., 2009) and exercise (Felip et al., 2013). Hepatic conversion of glucose to glycogen is considered a key step in defining metabolic flexibility toward dietary CHO utilization (Rito et al., 2018). Glucose conversion to glycogen can occur by the so-called direct pathway (glucose – glucose-6-phosphate – glucose-1-phosphate – UDP-glucose – glycogen). Alternatively, glucose can be initially metabolized to the 3-carbon intermediate pyruvate, followed by gluconeogenic conversion back to the 6-carbon metabolite glucose-6-phosphate (G-6-P), known as the indirect pathway (Newgard et al., 1983; Figure 1). The indirect pathway mainly provides a means of synthesizing glycogen from non-glucose precursors such as glycerol and the catabolism of gluconeogenic amino acids. For carnivorous fish that are grown on conventional feeds high in protein and fish oil, these substrates are far more abundant than glucose (Viegas et al., 2012). Thus, as dietary protein is replaced by starch, the relative contribution of direct and indirect pathways to hepatic glycogen synthesis reflects the capacity for postprandial hepatic glucose disposal via the direct pathway versus utilization of gluconeogenic precursors via the indirect pathway. Measurement of direct and indirect pathway activities have been developed using novel stable isotope tracer methodologies, that are highly practical for juvenile fish raised in small recirculating systems in the research setting (Viegas et al., 2012, 2015, 2016; Martins et al., 2013; Rito et al., 2018, 2019).

In European seabass fed with archetypal high protein diet, direct pathway contributions were minimal and almost all glycogen synthesis was accounted for by the indirect pathway. When dietary protein was replaced by digestible starch, there was a significant reduction in indirect pathway contributions indicating a certain degree of metabolic flexibility toward the assimilation of dietary starch (Viegas et al., 2015). Importantly, hepatic glycogen levels *per se* were similar for the fish fed each diet hence this parameter did not inform the capacity for starch utilization under these study conditions.

In addition to the redirection of hepatic glycogen synthesis toward the direct pathway induced in seabass fed high starch diets (Viegas et al., 2015), we recently demonstrated this was accompanied by significant alterations in muscle metabolome (Jarak et al., 2018). We hypothesized that if barramundi had the metabolic flexibility to induce the conversion of dietary starch into glycogen via the direct pathway, this would also be associated with a unique pattern of changes in tissue metabolome. To test this hypothesis, we measured direct and indirect pathway contributions to hepatic glycogen synthesis using deuterated water (^2^H_2_O) in barramundi fed standard or high starch diets, and analyzed the hepatic and muscle metabolome. A combination of ^1^H and ^2^H NMR was employed to assess their sensitivity and specificity to alterations in hepatic glycogen synthesis.

## Materials and Methods

### Animal Welfare Disclaimer

All experiments were performed in accordance with the Australian code of practice for the care and use of animals for scientific purposes and were approval by the CSIRO Animal Ethics Committee (approval numbers: A8-2016).

### Fish Handling and Sampling

Tissue from a previous study were used in this experiment (Viegas et al., 2019). Briefly, two groups of 30 juvenile barramundi (*L. calcarifer*) (51.4 ± 0.5 g) were housed in a recirculated seawater system (200 L; 28 ± 1°C temperature; 35 ± 1‰ salinity; 7 mg L^–1^ dissolved O_2_). Each group was assigned one of the two experimental diets and were fed once daily to satiety, for 21 days. The diets were based on previous studies (Glencross et al., 2014) and formulated to vary relative contributions of protein or starch while maintaining the same digestible energy: Protein (P) – with high protein content (no digestible starch); and Starch (S) – with high digestible (pregelatinized) starch content (Table 1). Fish were then transferred into ∼3.5% ^2^H-enriched seawater by adding 99.9% deuterated water (^2^H_2_O; Sigma cat. #151882), as previously described (Viegas et al., 2011). Fish were kept in ^2^H_2_O for a total of 6 days, being fed to satiety once daily for 5 days and sampled 24 h after last meal. Fish were anesthetized in Aqui-S^®^, measured, weighed, and blood samples were collected from the caudal vein with heparinised syringes. Fish were euthanized by overdose in the same anesthetic; liver was excised and weighed. Hepatosomatic index (HSI) was calculated as: liver weight/body weight × 100. White muscle was collected from the epaxial quadrant (dorsal shoulder, above the midline) and weighed. Samples were stored at −80°C until further analysis.

**TABLE 1:** Formulation, proximate composition, digestible protein and energy parameters of the diets [Diet P – with high protein content (no digestible starch); Diet S – with high digestible, pregelatinized starch content].

	Diet P	Diet S
*Formulation*		
Fishmeal^1^	640	560
Gluten^2^	100	100
Casein^3^	100	50
Fish Oil^1^	40	0
Pregelatinized starch^2^	0	240
Yttrium Oxide	2	2
Vitamins and minerals^4^	5	5
Cellulose^3^	113	43
*Composition*		
Dry matter (DM)	930	890
Crude protein	633	502
Digestible protein	575	448
Total lipid	117	66
Ash	90	115
Total carbohydrates	161	317
Total starch	16	325
Gross energy (kJ g^–1^ DM)	21.3	20.8
Digestible energy (kJ g^–1^) DM)	15.9	15.2
Protein energy (%)	78	66
Lipid energy (%)	19	11
Starch energy (%)	0	20

*All values are g kg^–1^ dry matter (DM) basis unless stated otherwise. ^1^Peruvian anchovetta fishmeal and fishoil: Skretting Australia, Cambridge, TAS, Australia. ^2^Wheat gluten and pregelatinized wheat starch: Manildra, Auburn, NSW, Australia. ^3^Vitamin-free casein and Cellulose: Sigma, St. Louis, Missouri, United States. ^4^Vitamin and mineral premix includes (IU kg^–1^ or g kg^–1^ of premix): Vitamin A, 2.5 MIU; Vitamin D3, 0.25 MIU; Vitamin E, 16.7 g; Vitamin K, 3, 1.7 g; Vitamin B1, 2.5 g; Vitamin B2, 4.2 g; Vitamin B3, 25 g; Vitamin B5, 8.3; Vitamin B6, 2.0 g; Vitamin B9, 0.8; Vitamin B12, 0.005 g; Biotin, 0.17 g; Vitamin C, 75 g; Choline, 166.7 g; Inositol, 58.3 g; Ethoxyquin, 20.8 g; Copper, 2.5 g; Ferrous iron, 10.0 g; Magnesium, 16.6 g; Manganese, 15.0 g; Zinc, 25.0 g.*

### Sample Processing and Metabolite Extraction

Blood samples were centrifuged (3000 × *g*, 10 min), and plasma was stored for quantification of body water ^2^H-enrichment. Both liver and muscle samples of five fish were pooled into six replicate groups (pooled extraction/analyses: *n* = 6 per diet). Due to the lower sensitivity offered by NMR spectroscopy, this was designed to insure that yields for ^1^H and ^2^H NMR spectra contained high signal-to-noise ratios. This was particularly important for the detection of excess ^2^H-enrichment in hepatic glycogen, which has been described to have slow turnover rates in carnivorous fish (Viegas et al., 2012). Samples were ground in N_2_ and tissue metabolites extracted following the MTBE method as described previously (Matyash et al., 2008). This method allows the precipitation of insoluble residue (containing glycogen) and aqueous and organic phase separation. Each were separated, transferred to new vials and lyophilized to eliminate residual solvents. Dried samples were kept at room temperature until further analysis. The aqueous phase was ready for ^1^H NMR acquisition. Glycogen was extracted from the insoluble residue resulting from the MTBE extraction by alcoholic precipitation after alkaline hydrolysis. For ^2^H NMR acquisition with optimized the signal resolution, glycogen was further hydrolyzed to its glucosyl units using amyloglucosidase from *Aspergillus niger* (Glucose-free preparation, Sigma-Aldrich, Germany) and derivatized to monoacetone glucose (MAG) as described previously in detail (Viegas et al., 2012). An aliquot was kept for liver glycogen quantification, which was performed, in a fully automated analyzer Miura 200 (I.S.E. S.r.l.; Guidonia, Italy) using a dedicated glucose reagent kit (ref. A-R0100000601; *n* = 6).

### ^2^H NMR Acquisition

Proton-decoupled ^2^H NMR spectra were obtained with a Bruker Avance III HD 500 spectrometer using a ^2^H-selective 5 mm probe incorporating a ^19^F-lock channel. To quantify fish body water (BW) and tank seawater (TW) ^2^H-enrichments, 10 μL samples were added to 450 μL of calibrated acetone as previously described (Jones et al., 2001) with the addition of 50 μL of hexafluorobenzene (Sigma-Aldrich H8706, Spain) to detect ^19^F-lock (analyzed in duplicate). MAG samples were reconstituted in 0.5 mL 90% acetonitrile/10% ^2^H-depleted water with the addition of 50 μL of hexafluorobenzene. ^2^H NMR spectra were obtained following the acquisition parameters described in Rito et al. (2018).

### Estimation of Hepatic Glycogen Sources From ^2^H_2_O

Fish glucosyl units synthesized to glycogen during the 6-days residence in ^2^H_2_O become enriched in specific positions as a result of water hydrogen exchange and/or addition to metabolic intermediates catalyzed by specific enzymes of the glycogen synthesis pathways. Their positional ^2^H-enrichments were determined using the MAG methyl signals (MAG-CH_3_) as intramolecular standard (Nunes and Jones, 2009) and used to estimate the fractional metabolic provenance relative to tank water (Rito et al., 2018). The metabolic model for positional ^2^H-labeling is schemed in Figure 1 and a representative spectrum from which data is presented in Supplementary Figure S1. All indirect pathway contributions are calculated from the ^2^H-enrichment in position 5 (^2^H5) relative to tank water as follows:

(1) All indirect pathway contributions (%)=100×H2⁢5/TW

Glycogen synthesized from Krebs cycle precursors, is enriched in both positions 5 and 6 while glycogen derived from substrates that enter glycogenesis at the level of triose phosphates (Triose-P), such as glycerol released via lipolysis, is only enriched in position 5. Thus, indirect pathway contributions from the Krebs cycle are calculated from the ^2^H-enrichment in position 6*_*S*_* (^2^H6*_*S*_*) relative to tank water as follows:

(2) Contribution from all Krebs cycle substrates (%) =100×H26/STW

Contributions from Triose-P are estimated as the difference between position 5 and 6*_*S*_* enrichments relative to tank water:

(3) Contribution from Triose-P (%)=100×(2H5-2H6)S/TW

The remaining glycogen was either pre-existing, had undergone cycling via G-6-P, or was derived from the direct pathway. This contribution was calculated as follows:

Pre-existing+cycled+contribution from direct pathway (%)

(4)  =100-All indirect pathway contributions

All spectra were processed in the ACD/NMR Processor Academic Edition from ACD\Labs 12.0 software (Advanced Chemistry Development, Inc.) applying: zero-filling to 65 k, line broadening of 1.0 Hz, phasing and baseline correction. Parameters were compared between diets using Mann-Whitney test in GraphPad Prism software (GraphPad Software, La Jolla, CA, United States). Differences were considered statistically significant at *p* < 0.05.

### ^1^H NMR Acquisition

Water-soluble metabolites from liver and muscle were reconstituted in a 50 mM phosphate buffer (pD = 7.4) in ^2^H_2_O (99.8%, CortecNet, France), with 4.94 mM 3-(trimethylsilyl) propionic-2,2,3,3-d4 acid sodium salt (TSP) (Sigma-Aldrich, Spain) as an internal NMR standard. Proton-decoupled ^1^H NMR spectra were obtained using a Varian VNMRS 600 MHz (Agilent, Santa Clara, CA, United States) spectrometer equipped with a 3 mm ^1^H(X)-PFG inverse configuration probe. A CPMG pulse sequence was used for each liver sample (spectral width 7 kHz; acquisition time 3 s; saturation delay 2 s; relaxation delay 2 s; 32 scans; 512 ms echo- time; at 298 K). For the muscle samples a ^1^H-Presat pulse sequence was used (spectral width 7 kHz; acquisition time 4.45 s; saturation delay 3 s; relaxation delay 4 s; 32 scans; at 298 K). To assist metabolite identification, a homonuclear J-Resolved spectrum was collected on a representative sample of each group, using a standard Varian pulse sequence. All spectra were processed in the ACD/NMR Processor Academic Edition from ACD\Labs 12.0 software (Advanced Chemistry Development, Inc.) applying: zero-filling to 65 k, line broadening of 0.2 Hz, phasing and baseline correction. The chemical shifts were referenced to TSP peak at 0 ppm.

### Spectral Analysis – Targeted Analysis

Metabolites were identified by querying the Human Metabolome Database, the library of Chenomx NMR suite V8.3 (Chenomx Inc., Edmonton, AB, Canada) and the online tool MetaboHunter (Tulpan et al., 2011). Metabolite relative quantification was determined by peak integration on selected peaks for each metabolite using ACD\Labs 12.0 Software. Variation between the groups was expressed as fold change (FC = relative concentration in S group/relative concentration in P group); and compared using Student’s *t*-test or Mann-Whitney test, according to its conformity to normal assumptions. Univariate analyses were performed in GraphPad Prism software (GraphPad Software, La Jolla, CA, United States). Differences were considered statistically significant at *p* < 0.05. Metabolites have been identified at Metabolomics Standards Initiative (MSI) level 2 according to the guidelines for metabolite identification (Sumner et al., 2007). The metabolomics data generated during the current study were submitted to the EMBL-EBI MetaboLights database with the identifier MTBLS1103^1^.

### Spectral Analysis – Untargeted Analysis

Spectral binning was performed in ACD/NMR Processor Academic Edition from ACD\Labs 12.0 software, using uniform binning with 0.04 ppm width, from −0.5 to 10 ppm. Regions for water (4.51 to 5.52 ppm) and TSP (−0.29 to 0.06 ppm) were excluded. Multivariate analysis was applied to evaluate the general group separation of liver and muscle samples. Principal component analysis (PCA) and partial least squares discriminant analysis (PLS) were performed on the online software MetaboAnalyst 3.0^2^, using the spectra buckets integrals values. For the PLS, Q^2^ (predictive ability of the model), R^2^ (goodness of the fit), and the *p*-value of the permutation test (1000 permutations) were considered as quality parameters of the model. Models were accepted as valid for Q^2^ above 0.5 and *p*-value < 0.05. All ellipses in the scores plots, for both PCA and PLS models, were drawn at the 95% confidence level.

## Results

### Hepatic Glycogen

**FIGURE 2:**
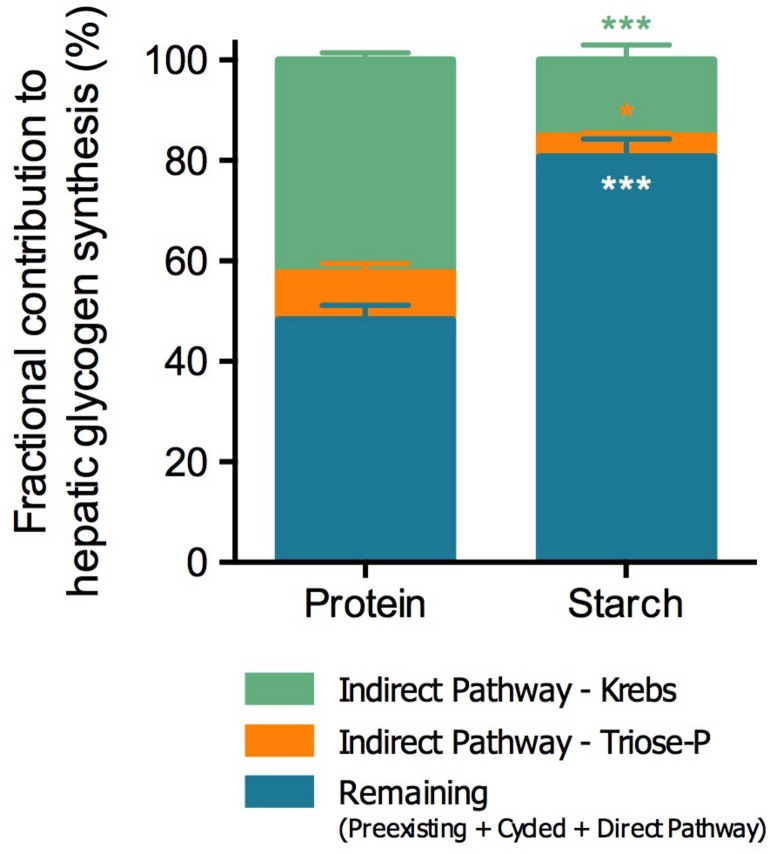
Fractional contribution to hepatic glycogen synthesis of barramundi (*L. calcarifer*) fed with high protein or high starch diets. Contributions are represented as percentage of total (%). Values are mean ± SEM (*n* = 6). Significant differences are indicated by asterisks (Mann-Whitney test; *p < 0.05, **p < 0.01, and ***p < 0.001). The same color scheme was used as in Figure 1.

Average tank water (TW) ^2^H-enrichment was 3.6 ± 0.2% (mean ± SEM), which was consistent with the respective fish body water (BW) (Protein: TW 3.46 ± 0.19 vs. BW 3.25 ± 0.09; Starch: TW 3.79 ± 0.04 vs. BW 3.74 ± 0.12). The hepatosomatic index (HSI) was lower in fish fed with the Starch diet (Protein 1.88 ± 0.05 vs. Starch 1.30 ± 0.07; *t*-test *p* < 0.001), while total hepatic glycogen content did not differ between diets (Protein: 7.5 ± 0.5 vs. Starch: 6.9 ± 0.3 g 100 g^–1^ liver; *t*-test *p* > 0.05). However, the analysis of the sources of hepatic glycogen revealed significant differences between groups (Figure 2). In the Starch-fed fish, the overall contribution of the indirect pathway was significantly decreased from 52% to 19% of total glycogen. This decrease in indirect pathway contributions was proportionally distributed between Krebs cycle sources, which fell from 42% to 15%, and Triose-P sources such as glycerol, which decreased from 9% to 4%.

### Liver and Muscle Aqueous Metabolome

**FIGURE 3:**
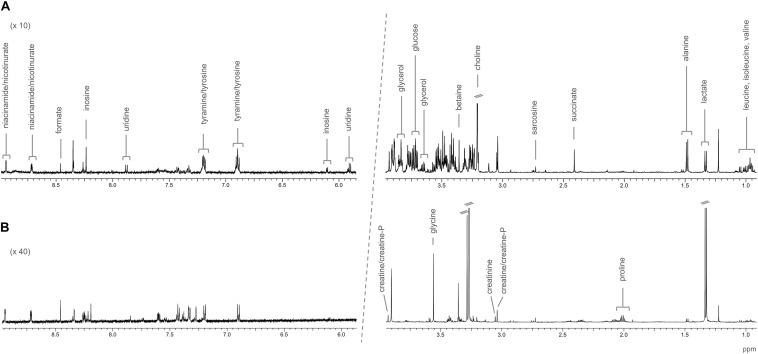
Representative NMR spectra of barramundi (*L. calcarifer*), with the assignments of identified metabolites; **(A)** CPMG spectrum of the aqueous fraction of liver; and **(B)** 1D ^1^H spectrum of the aqueous fraction of muscle.

**FIGURE 4:**
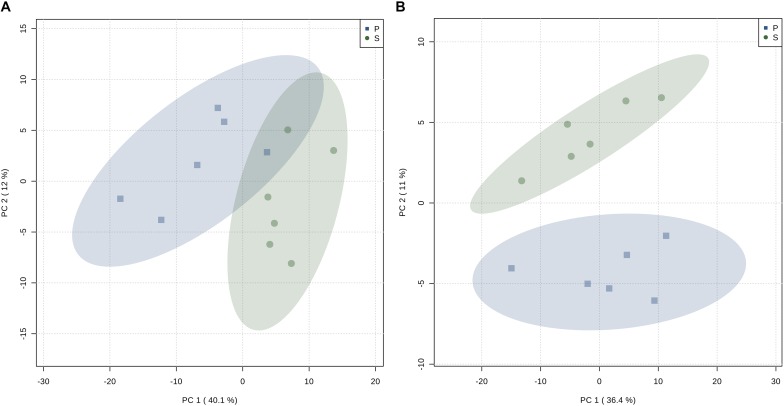
Principal Component Analysis (PCA) scores plot of: **(A)** CPMG spectra of the liver aqueous fraction, and **(B)** 1D ^1^H NMR spectra of the muscle aqueous fraction of barramundi (*L. calcarifer*) fed with high protein (P) or (S) high starch diets.

**FIGURE 5:**
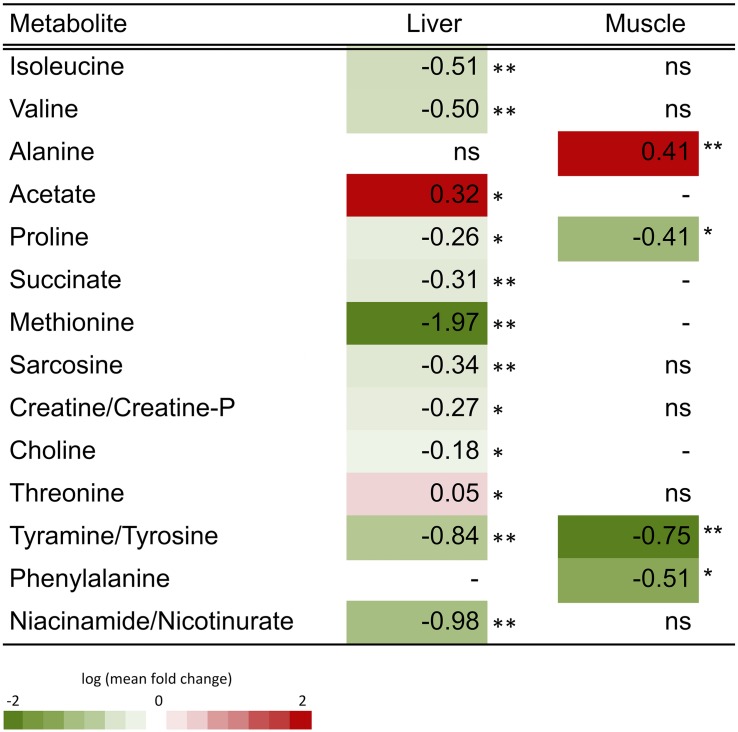
Heatmap of the fold-change variation (log mean FC, mean relative concentration in S group/mean relative concentration in P group) of the metabolites identified in the aqueous fraction of liver and muscle of barramundi (*L. calcarifer*). Student’s *t*-test or Mann-Whitney test were applied dependent on the conformity to normal distribution. Detailed information about all metabolites is presented in supplementary information (Supplementary Tables S1, S2). Key: (–) metabolite not identified in the tissue; ns, fold change with non-significant variation; ^∗^*p* < 0.05, ^∗∗^*p* < 0.01, and ^∗∗∗^*p* < 0.001.

Representative spectra of the liver and muscle aqueous fractions are shown in Figure 3. Assignments of the metabolites identified in both tissues are provided as Supplementary Material (liver: Supplementary Table S1, muscle: Supplementary Table S2). Multivariate analysis of liver and muscle samples are presented in Figure 4. The PCA plot of liver metabolite data presented an incomplete separation between groups (Figure 4A), with the majority of the samples separated along the first principal component (PC1) that explained 40.1% of the variation between the two diets. PCA analysis of the muscle NMR spectra (Figure 4B) showed a complete separation between fish fed the S or P diets, which occurred exclusively along the PC2 axis that represented 11% of the variation between the two diets. We identified the spectral signatures of 22 water-soluble metabolites in the liver and muscle aqueous fractions (liver: Supplementary Table S1, muscle: Supplementary Table S2). Metabolites with statistically significant variations between fish fed the S diet relative to the P diet are presented in Figure 5. In liver, only acetate and threonine were significantly higher in fish fed the S diet compared with the P diet. Meanwhile, the metabolites methionine, niacinamide/nicotinurate and tyramine/tyrosine were significantly reduced in fish fed the S diet. For the muscle samples, there were fewer water-soluble metabolites that showed significantly different abundances between fish fed the S or P diets. Alanine was the only metabolite that was higher in fish fed the S diet, while the levels of tyramine/tyrosine, phenylalanine and proline were all reduced in fish fed the S diet. PLS models did not fulfill the validation tests required to attribute significant differences between diets, so were not presented.

## Discussion

### Glycogen Synthesis via the Direct Pathway as a Proxy for Effective Starch Utilization by Carnivorous Fish

The combined ^1^H NMR-based metabolomics and ^2^H NMR analysis of excess enrichment in hepatic glycogen of barramundi fed with a high-starch diet provided evidence of the mechanisms for starch utilization in this species. The general consensus is that dietary glucose utilization by carnivorous fish is limited, for barramundi in particular (McMeniman, 2003; Glencross, 2006; Glencross et al., 2012; Wade et al., 2014). However, this study provided evidence that barramundi have the capacity to absorb and metabolize dietary CHO in the liver, to the extent that dietary sources were preferred over gluconeogenic sources for hepatic glycogen synthesis (Figure 1). By sustaining endogenous glucose levels and the rate of hepatic glycogen synthesis, CHO-enriched diets could in principle decrease the requirement for endogenous synthesis of glucose-6-phosphate (G-6-P). This molecule is the central precursor for glucose, glycogen and glucosyl moieties of glycoproteins, especially abundant in fish mucus. Carnivorous fish such as the European seabass normally synthesize glycogen almost entirely via the indirect pathway (Viegas et al., 2012, 2015), although seabass showed evidence of direct pathway induction by dietary CHO (Viegas et al., 2015), which was consistent with our current findings in barramundi. This demonstrates that dietary glucose absorption, and its subsequent conversion to G-6-P in the liver, is sufficient to substantially displace glycogen synthesis from gluconeogenic precursors. In contrast to other studies of CHO utilization by carnivorous fish, the metabolic adaptations observed in barramundi was not accompanied by significant changes in hepatic glycogen levels. When subjected to high dietary CHO, carnivorous species like European seabass (Enes et al., 2011; Castro et al., 2015), gilthead seabream (Enes et al., 2011; Ekmann et al., 2013), Senegalese sole (*Solea senegalensis*) (Conde-Sieira et al., 2016), and rainbow trout (*Oncorhynchus mykiss*) (Polakof et al., 2009, 2010; Kamalam et al., 2012) or herbivorous species like gibel carp (*Carassius auratus*) and grass carp (*Ctenopharyngodon idellus*) (Tian et al., 2012; Li et al., 2016) generally display increased HSI and glycogen levels in liver and/or muscle, or as whole fish content. Thus, while increases in hepatic or systemic glycogen levels may serve as markers for CHO utilization, our study demonstrates that significant metabolic adaptations to CHO can occur without influencing total glycogen levels. At the same time, the HSI was significantly lower with the Starch diet which can be revealing of implications for hepatic lipid metabolism. These are expected since part of starch-supplementation was made at the expense of fish oil and include increased levels and faster turnover of hepatic triacylglycerol through increased *de novo* lipogenesis (Wade, unpublished). From a methodological perspective, the use of stable isotopes other than ^2^H (e.g., ^13^C-starch in custom-made diets) may allow the fate of specific dietary substrates to be followed, and discriminate between the different metabolic pathways required to fulfill the physiological needs of the fish. In gilthead seabream fed increasing levels of starch (equally ^13^C-enriched), there was a continuous increase of ^13^C-enrichment in glycogen pool was observed, in both liver and whole fish tissues (Ekmann et al., 2013). The liver displayed the highest enrichment response and approximately one-third of the hepatic glycogen originated from sources other than dietary starch (indirect pathway), which was consistent with the findings described here for barramundi using ^2^H. It is worth noting that ^13^C-glycogen may have been derived from ^13^C-glucose previously metabolized and resynthesized via gluconeogenesis. In gilthead seabream fed with ^13^C-starch, exercise did not alter hepatic glycogen levels nor the ^13^C-enrichment pattern between 6 and 24 h after feeding (Felip et al., 2013). In rainbow trout, the pattern of incorporation of the ^13^C-enrichment into different compounds was influenced by the type of starch, where fish fed with raw starch the highest amount of ^13^C recovered in liver was contained in protein (36%) > glycogen (32%) > lipids (8%), while fish fed with gelatinized starch the highest ^13^C labeled fraction was lipids (27%) > glycogen (19%) > protein (12%). Even so, differences between diets were not observed (Felip et al., 2012). These studies emphasize the importance of glycolysis and the effect of dietary gluconeogenic precursors and nutrient ratios, as a means to assess and optimize fish metabolism. In addition to dietary stimuli or exercise, hepatic glycogen is highly responsive to food deprivation and other types of stress, most likely through hormone-triggered mechanisms (Polakof et al., 2011). Such mechanisms are largely unexplored in barramundi, however, the use of [^14^C]radioisotope studies revealed that IGF-I and insulin stimulated the use of glucose for muscle glycogen synthesis in this species (Drakenberg et al., 1997; Degger et al., 2000). Another study in barramundi revealed decreased serum IGF-I levels resulted from a diet with an increased (unbalanced) carbohydrate/lipid ratio of non-protein energy (Nankervis et al., 2000). These data highlight the cross-talk that occurs between nutrients and hormonal action, as observed for rainbow trout (Polakof et al., 2011; Dai et al., 2015; Marandel et al., 2016).

### The Liver and Muscle Metabolome Reflects Hepatic Glycogen Synthesis Capacity

Multivariate analysis showed that both liver and muscle metabolite composition was modified by diet. In the liver, clustering occurred mainly along the first principal component, which in this case explains the variation between the experimental groups (40.1%). On the other hand, the muscle samples grouped separately, but the variation between experimental groups is only explained by the second principal component (11%). So, although the liver had an incomplete sample separation, its multivariate model explained more of the variation between groups than the model for the muscle samples. Univariate analysis also revealed variations between groups, particularly in liver. The quantitative change of each metabolite in muscle and liver was then essential to evaluate the response to the dietary starch. In the liver, methionine (Met) was the most significantly reduced metabolite after ingestion of high starch diets. Met is an essential proteinogenic amino acids required for growth and a source of metabolic intermediates. Methionine is an important methyl donor for several reactions such as the biosynthesis of polyamines, L-carnitine and cysteine (Cys), and DNA methylation. Moreover, methionine can scavenge ROS from the cells and acting then as antioxidant (Coutinho et al., 2017) and affect folate (vitamin B9) cycle (Tardy et al., 2020). Decreased values of Met in the fish fed the S diet could imply an unbalanced status of the liver. Often considered the first rate limiting essential amino acid, DL-methionine is commonly added to plant based diets that contain sub-optimal levels. Such supplementation in barramundi revealed greater utilization of Met from dietary fishmeal than from a lupine-based diet supplemented with DL-methionine (Poppi and Glencross, 2018). Similarly, markers of sulfur amino acid turnover in barramundi were more significantly affected by time after feeding than by dietary Met level (Poppi et al., 2018), suggesting a limited effect of dietary composition. Other sulfur amino acids Cys and taurine (Tau) can also substitute for dietary Met, but Cys was not detected in our hepatic metabolome while Tau levels were unaffected by diet. Dietary Met can be metabolized to sarcosine, which could explain its reduction in the liver of individuals fed S diet. Although requiring further investigation, the present data supports the idea that a proportion of dietary Met from plant-based ingredients was used for processes other than protein deposition, possibly catabolized for energy or excreted. Results also reinforce the idea that Met is a key nutrient for carnivorous fish, suggesting that its dietary inclusion should be increased along with the starch content.

Niacinamide and nicotinurate molecules are not well resolved by ^1^H NMR. However, niacinamide is the active form of niacin (vitamin B3) and precursor of nucleotides and NAD(P), responsible for electron transport in hydrogen complexes, thus allowing interpretation for this metabolite. Depletion of these enzymatic complexes could be linked to changes in energetic status, in *de novo* lipogenesis and antioxidant activity in the liver (Ng et al., 1997; Segner et al., 1997). The study performed in parallel with the same animals and following the current experimental design revealed no differences in the muscle fatty acids composition and lipogenic flux of fish fed a high starch diet. However, it was indeed reported an increase in the *de novo* lipogenesis in the visceral adipose tissue fish fed the high starch diet (Viegas et al., 2019).

A significant increase in the relative concentration of muscle alanine (Ala) was observed in Starch fed fish. Alanine can be easily biosynthesized from pyruvate and other branched chain amino acids such as valine and isoleucine, which were also significantly reduced in liver. Alanine is then a key intermediate in glycolysis, gluconeogenesis and Krebs cycle. As intermediate of the Cahill cycle, an increase in muscle was associated with favoring of the glycolytic pathway in mammals (O’Donnell et al., 2004). Although no differences were observed in the other intermediates of the cycle, this result can be indicative of a decrease in the alanine exchange between muscle and liver, or a disruption of the cycle. However, without additional information about the enzymatic activity or other intermediates of the cycle, it is limitative to draw further conclusion. A previous study on European seabass fed a raw starch diet also found alanine increased in muscle (Jarak et al., 2018) and a resulting decrease on the daily growth index (Viegas et al., 2016). The data presented here further implicate Ala in the metabolic adjustments to high starch diets in fish that also favor glycolysis over gluconeogenesis. Other amino acids also showed significant changes in the liver and muscle of S group fish. We noted that the abundance of dietary amino acids in the S diet was 71–88% that of the P diet, which translates to a log fold decrease of -0.1. Therefore, the dietary amino acid levels were not the principal driver of the differences in S and P metabolite profiles. Amino acid levels contained within the two diets were balanced, and did not reveal any major deficiencies in nutrient supply (Glencross et al., 2014). However, differential utilization of amino acids from different dietary ingredients is well known (Williams et al., 2001; Glencross, 2006) and might have contributed to the differences in hepatic amino acid concentrations observed in this study. Although results from hepatic glycogen synthesis are indicative of a clear reduction of the indirect pathway, it seems that some amino acids are still utilized as gluconeogenic precursors. However, these amino acids seem to come from other source other than the fish muscle, since few amino acids identified in muscle had decreased concentrations in S group, although this group had lower body gain (Glencross B. D. et al., 2017). It is possible that amino acids were provided by diet, since fish fed high starch diet had higher feed intake (Glencross B. D. et al., 2017) and directly utilize for energy production rather than muscle growth. The relative concentrations of tyrosine, isoleucine, valine and proline significantly decreased, suggesting the utilization of these amino acids by fish fed diets with high starch content. Tyrosine kinase activity in fish is also affected by insulin levels, which was shown to be depressed in fish fed starch-enriched diets as affected by the dietary nutrients ratio (Kamalam et al., 2012). Both isoleucine and valine can be used in Krebs cycle or gluconeogenesis when the carbohydrates are reduced or unavailable in the diet (Prathomya et al., 2017). The same trend of variation was observed for isoleucine, valine and proline in muscle of European seabass fed with high digestible starch diet (Jarak et al., 2018). Together with these amino acids, also alanine varied in the same trend in the present work and the previous study by Jarak et al. (2018). These carnivorous species seem to response in similar ways when subjected to diets with higher starch percentages.

## Conclusion

Results clearly revealed that hepatic glycogen synthesis was shifted toward the direct pathway by the starch-enriched diet. Starch utilization can potentially spare the catabolism of dietary amino acids to glucose and glycogen, avoiding needless nitrogenous waste. This can ultimately improve water quality while yielding other environmental benefits. This metabolic adaptation is supported by key changes in the muscle and liver metabolome, such as the increase in muscular alanine. Although metabolic results are indicative of an increase in the indirect pathway, it seems that some amino acids are still being utilized as gluconeogenic precursors. However, since few amino acids identified in muscle had decreased concentrations in the group fed high starch diet, it is highly unlikely that these amino acids were sourced from the fish muscle. Muscle tissue, as the main product of interest for aquaculture, was less affected as already reported for its fatty acid composition (Viegas et al., 2019), but also as regards to its metabolite composition.

## Data Availability Statement

The metabolomics datasets generated during the current study were submitted to the EMBL-EBI MetaboLights database with the identifier MTBLS11031 (https://www.ebi.ac.uk/metabolights/MTBLS1103). Other datasets generated for this study are available on request to the corresponding author.

## Ethics Statement

The animal study was reviewed and approved by CSIRO Animal Ethics Committee (approval number: A8-2016).

## Author Contributions

IV and NW designed the experiments. IV, JJ, BG, and NW provided funding for material and instrumentation to conduct the work. LTr and NW conducted the experiments and pre-processed samples. MP, LTa, JR, and ES processed and analyzed samples. MP, LTa, NW, JJ, and IV discussed and interpreted the results. MP, NW, and IV wrote the manuscript with everyone’s contributions. All authors reviewed the manuscript.

## Conflict of Interest

The authors declare that the research was conducted in the absence of any commercial or financial relationships that could be construed as a potential conflict of interest.

## Footnotes

1 https://www.ebi.ac.uk/metabolights/MTBLS1103

2 https://www.metaboanalyst.ca
