# Supplementary material for: Limitations to Starch Utilization in Barramundi (Lates calcarifer) as Revealed by NMR-Based Metabolomics
Source: Front Physiol. 2020 Mar 20;11:205. doi: 10.3389/fphys.2020.00205 (PMC7098972; doi:10.3389/fphys.2020.00205)
Supplement: Supplementary file 1 [file Data_Sheet_1.pdf]

Supplementary Material

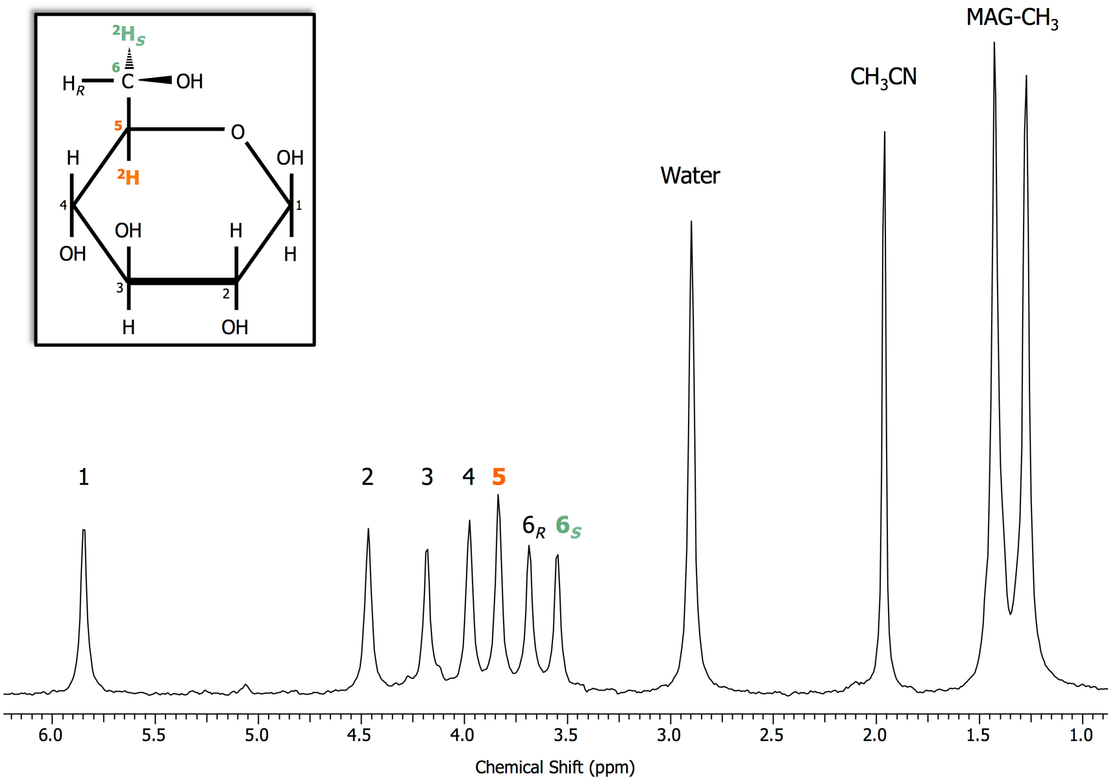

Supplementary Figure S1 - ^{2}H NMR representative spectra of monoacetone glucose (MAG) samples derived from liver glycogen of barramundi (*L. calcarifer*) sampled after 6-day residence in a tank with 3.5% 2H-enriched seawater. The numbers above each signal represents its position within the original glucose molecule (inlet). The same colour code was used as in Fig. 1 positional enrichments: 5 (orange) and 6_S (green). Other signals include water and acetonitrile (CH_3CN) as solvents, and the pair of ^{2}H-enriched methyl signals used as internal ^{2}H enrichment references (MAG-CH_3)

Supplementary Table S1 - Identified metabolites in the liver aqueous fraction of barramundi (*L. calcarifer*), with its assigned peaks, the compound identification code (ChEBI), and the fold-change variation (log mean FC = relative concentration in S group/relative concentration in P group mean FC). Student's t-test or Mann-Whitney test were applied dependent on the conformity to normal assumptions. Key: (s) singlet, (d) doublet, (t) triplet, (m) multiplet, (dd) double doublets, (*) $p < 0.05$; (**) $p < 0.01$, (§) peaks selected for integration.

Metabolites (ChEBI)	Assignments	ppm (multiplicity)	log(FC)	
Isoleucine (24898)	δ-CH_3, δ-CH_3, α-CH	0.94 (t)§, 1.01 (d)§, 3.68 (d)	-0.51	**
Valine (27266)	γ-CH_3, α-CH	0.99 (d)§, 3.61 (d)	-0.50	**
Lactate / Threonine (78320/26986)	CH3, CH / CH, α-CH	1.3 (d)§, 4.1 (dd) / 1.3 (d), 3.60 (m)	-0.06	
Alanine (16449)	CH_3, CH	1.48 (d)§, 3.78 (q)	-0.05	
Acetate (15366)	CH_3	1.92 (s)§	0.32	*
Proline (17203)	4-CH_2, 3-CH_2, 3-CH_2	1.97-2.10 (m), 2.06- 2.10 (m)§, 2.30-2.37 (m)	-0.26	*
Succinate (15741)	CH_2	2.41 (s)§	-0.31	**
Methionine (16811)	β-CH_2, β-CH_2, CH_3, γ-CH_2	2.07-2.14 (m), 2.16-2.22 (m), 2.14 (s), 2.65 (s)§	-1.97	**
Sarcosine (15611)	CH_3	2.73 (s)§	-0.34	**
Creatine/Creatine-P (16919/17287)	CH_3, CH_2	3.04 (s)§, 3.92 (s)	-0.27	*
Creatinine (16737)	CH_3	3.05 (s)§	-0.05	
Choline (15354)	CH_3	3.21 (s)§	-0.18	*
Betaine (17750)	CH_2	3.27 (s)§	-0.08	
Taurine (15891)	β-CH_2, α-CH_2	3.28 (t)§, 3.42 (t)	0.01	
Threonine (26986)	CH, α-CH	1.3 (d), 3.60 (m)§	0.05	*
Glycerol (17754)	CH_2, CH	3.64 (m)§, 3.78 (m)	0.01	
Glucose (17634)	CH	3.22-3.25 (m), 3.37-3.40 (m), 3.70-3.74 (m)§	-0.08	
Tyramine/Tyrosine (15760/17895)	3',5'-CH, 2',6'-CH	6.89 (d), 7.20 (d)§	-0.84	**
Uridine (16704)	1'-CH, 5-CH, 6-CH	5.92(d), 5.91 (d), 7.87 (d)§	-0.09	
Inosine (17596)	2-CH, 8-CH	8.22 (s)§, 8.35 (s)	0.07	
Formate (30751)	CH	8.46 (s)§	-0.02	
Niacinamide/Nicotinurate (17154/7563)	5'-CH, 6'-CH, 2'-CH	7.60 (dd), 8.71 (dd)§, 8.94 (d)	-0.98	**

Supplementary Table S2 - Identified metabolites in the muscle aqueous fraction of barramundi (*L. calcarifer*), with its assigned peaks, the compound identification code (ChEBI), and the fold-change variation (log mean FC = relative concentration in S group/relative concentration in P group mean FC). Student's t-test or Mann-Whitney test were applied dependent on the conformity to normal assumptions. Key: (s) singlet, (d) doublet, (t) triplet, (m) multiplet, (dd) double doublets, *p<0.05 and **p<0.01, (§) peaks selected for integration.

**Metabolites (ChEBI)**	**Assignments**	**ppm (multiplicity)**	**log(FC)**	
Valine (27266)	γ-CH_3, α-CH	0.99 (d)§, 3.61 (d)	-0.20	
Isoleucine (24898)	δ-CH_3, δ-CH_3, α-CH	0.94 (t)§, 1.01 (d)§, 3.68 (d)	-0.20	
Isobutyrate (16135)	CH_3	1.04 (d)§	-0.21	
Alanine (16449)	CH_3, CH	1.48 (d)§, 3.78 (q)	0.41	**
Proline (17203)	4-CH_2, 3-CH_2, 3-CH_2	1.97-2.10 (m)§, 2.06- 2.10 (m), 2.30-2.37 (m)	-0.41	*
β-Alanine (16958)	α-CH_2, β-CH_2	2.55 (t)§, 3.18 (t)	-0.30	
Sarcosine (15611)	CH_3	2.73 (s)§	0.19	
Dimethylglycine (17724)	1',2'-CH_3, CH_2	2.90 (s)§, 3.73 (s)	0.27	
Asparagine (17196)	CH_2	2.85 (dd), 2.94 (dd)§	-0.33	
Creatine/ Creatine-P (16919/17287)	CH_3, CH_2	3.04 (s)§, 3.93 (s)	0.12	
Carnitine (16347)	CH_3	3.20 (s)§	0.02	
Betaine (17750)	CH_2	3.27 (s)§	-0.02	
Glycine (15428)	CH_2	3.56 (s)§	0.01	
Threonine (26986)	CH, α-CH	3.59 (d)§, 4.26 (m)	-0.18	
Serine (17115)	CH, CH_2	3.84 (dd)§, 3.96 (m)	-0.28	
Lactate (78320)	CH_3, CH	1.32 (d), 4.10 (dd)§	-0.05	
Hydroxyproline (18095)	δ-CH_2, γ-CH	3.48-3.50 (m), 4.34-4.37 (m)§	-0.29	
Tyramine/ Tyrosine (15760/17895)	3',5'-CH, 2',6'-CH	6.89 (d)§, 7.20 (d)	-0.75	**
Phenylalanine (17295)	3',5'-CH	7.42 (t)§	-0.51	*
Inosine (17596)	2-CH, 8-CH	8.22 (s), 8.35 (s)§	-0.25	
Formate (30751)	CH	8.46 (s)§	0.15	
Niacinamide (17154)	5'-CH, 6'-CH, 2'-CH	7.60 (dd), 8.71 (dd)§, 8.94 (d)	-0.20	
